# Longitudinal Population Dynamics of *Staphylococcus aureus* in the Nasopharynx During the First Year of Life

**DOI:** 10.3389/fgene.2019.00198

**Published:** 2019-03-15

**Authors:** Shima M. Abdulgader, Lourens Robberts, Jordache Ramjith, Polite M. Nduru, Felix Dube, Sugnet Gardner-Lubbe, Heather J. Zar, Mark P. Nicol

**Affiliations:** ^1^Division of Medical Microbiology, Department of Pathology, Faculty of Health Sciences, University of Cape Town, Cape Town, South Africa; ^2^Division of Epidemiology and Biostatistics, School of Public Health and Family Medicine, University of Cape Town, Cape Town, South Africa; ^3^Department of Paediatrics and Child Health, Red Cross War Memorial Children’s Hospital and SA-MRC Unit on Child and Adolescent Health, University of Cape Town, Cape Town, South Africa; ^4^Department of Molecular and Cell Biology, Faculty of Science, University of Cape Town, Cape Town, South Africa; ^5^Department of Statistics and Actuarial Science, Stellenbosch University, Stellenbosch, South Africa; ^6^Institute of Infectious Disease & Molecular Medicine, Faculty of Health Sciences, University of Cape Town, Cape Town, South Africa; ^7^School of Biomedical Sciences, University of Western Australia, Perth, WA, Australia

**Keywords:** *Staphylococcus aureus*, carriage, infancy, genotype diversity, longitudinal

## Abstract

**Background:** *Staphylococcus aureus* colonization is a risk factor for invasive disease. Few studies have used strain genotype data to study *S. aureus* acquisition and carriage patterns. We investigated *S. aureus* nasopharyngeal carriage in infants in an intensively sampled South African birth cohort.

**Methods:** Nasopharyngeal swabs were collected at birth and fortnightly from 137 infants through their first year of life. *S. aureus* was characterized by *spa*-typing. The incidence of *S. aureus* acquisition, and median carriage duration for each genotype was determined. *S. aureus* carriage patterns were defined by combining the carrier index (proportion of samples testing positive for *S. aureus*) with genotype diversity measures. Persistent or prolonged carriage were defined by a carrier index ≥0.8 or ≥0.5, respectively. Risk factors for time to acquisition of *S. aureus* were determined.

**Results:** Eighty eight percent (121/137) of infants acquired *S. aureus* at least once. The incidence of acquisition at the species and genotype level was 1.83 and 2.8 episodes per child-year, respectively. No children had persistent carriage (defined as carrier index of >0.8). At the species level 6% had prolonged carriage, while only 2% had prolonged carriage with the same genotype. Carrier index correlated with the absolute number of *spa*-CCs carried by each infant (*r* = 0.5; 95% CI 0.35–0.62). Time to first acquisition of *S. aureus* was shorter in children from households with ≥5 individuals (HR 1.06, 95% CI 1.07–1.43), with *S. aureus* carrier mothers (HR; 1.5, 95% CI 1.2–2.47), or with a positive tuberculin skin test during the first year of life (HR; 1.81, 95% CI 0.97–3.3).

**Conclusion:** Using measures of genotype diversity, we showed that *S. aureus* NP carriage is highly dynamic in infants. Prolonged carriage with a single strain occurred rarely; persistent carriage was not observed. A correlation was observed between carrier index and genotype diversity.

## Introduction

*Staphylococcus aureus* colonizes the mucosal and epithelial surfaces of several body sites such as the nose, throat, gut, axilla, groin, vagina, and perineum. However, the nasal cavity is considered the primary anatomical site for colonization (Verhoeven et al., 2014). Asymptomatic carriage is a risk factor for infections, both community-acquired (particularly skin and soft tissue infections) and nosocomial (including surgical site infection, catheter-related infections, pneumonia, and endocarditis) (Sakr et al., 2018). Impairment in the local or systemic immune response, for example due to disruption of the skin barrier, extremes of age, or HIV infection are associated with progression to disease (Wertheim et al., 2005; Johannessen et al., 2012). Metabolic adaptation during lack of nutrients and enhanced expression of virulence genes which are mediated by two-component systems and global regulators play critical role in the transition of *S. aureus* from the colonizer to the invasive state (Pohl et al., 2009; Waters et al., 2016).

In adults, longitudinal studies describe three nasal carriage patterns based on the carrier index (the proportion of samples collected from each individual which are positive for *S. aureus*). Individuals with indices >0.8 or 1 are considered persistent carriers, individuals with an index of zero are non-carriers, and individuals with indices between 0 and 0.8 are considered intermittent carriers (Van Belkum et al., 1997; Vandenbergh et al., 1999; Blumental et al., 2013; Muthukrishnan et al., 2013; Verhoeven et al., 2014). Approximately one third of adults are considered to be persistent carriers (Wertheim et al., 2005). However, definitions of carriage are influenced by the frequency of sampling, the number of swabs collected and follow up period, which vary between studies (Van Belkum et al., 2009; Muthukrishnan et al., 2013; Verhoeven et al., 2014); seven swabs collected prospectively over 5 weeks were shown to distinguish *S. aureus* non-carriers from intermittent carriers (Nouwen et al., 2004).

Studies of *S. aureus* carriage in healthy populations reported higher persistent carriage rates among children compared to adults (Cunliffe, 1949; Noble et al., 1967; Armstrong-Esther and Smith, 1976). However, longitudinal studies of *S. aureus* nasal carriage patterns at the species level provide an incomplete description of the true nature of carriage as they are unable to distinguish between persistent carriage with a single strain and sequential carriage with different strains. True persistent colonization would imply carriage of a single genotype over an extended period (Peacock et al., 2003; Lebon et al., 2008). Few studies have used strain genotyping to support their definitions of persistent vs. intermittent nasal carriage, especially among infants (Blumental et al., 2013; Muthukrishnan et al., 2013; Schaumburg et al., 2013). Studies in children that have used strain genotyping, have only collected a very limited number of swabs over the period of study, and are therefore unable to accurately address this question (Blumental et al., 2013; Schaumburg et al., 2013).

In order to address this gap in knowledge, we therefore conducted intensive longitudinal NP sampling of a cohort of infants for 12 months and genotyped *S. aureus* strains using *spa*-typing. We determined, for the first time, *S. aureus* NP carriage dynamics (the incidence of *S. aureus* acquisition and number of carriage episodes) both at the species and the genotype levels. We show that whilst acquisition is common, persistent or prolonged carriage with the same genotype is rare in infants.

## Materials and Methods

### Study Population and Characterization of *S. aureus*

This study was nested within the Drakenstein Child Health Study – a population based cohort study of a 1143 mother-infant pairs based in a peri-urban community in Cape Town, South Africa (Zar et al., 2014). A total of 885 mother-infant pairs chose to enroll in an intensive cohort who were followed up fortnightly for the first year of life. Mothers were enrolled during the second trimester of pregnancy; details of the broader study are described elsewhere (Zar et al., 2014). Socioeconomic measures were from a composite validated score, HIV exposure was defined as HIV uninfected infants born to HIV-infected mothers, maternal, or paternal smoking was assessed by self-report. Tuberculin skin test (TST) reactivity was defined as ≥10 mm in HIV-uninfected children and ≥5 mm in HIV-infected children.

NP swabs (Copan flocked swab, FLOQSwabs^TM^, COPAN Diagnostics, Murrieta, CA, United States) were collected at birth from mother-infant pairs and fortnightly from infants through their first year of life. One hundred and thirty-seven mother-infant pairs were selected for this study – these were selected as the first infants to complete their first year of life, with a minimum set of 18 NP samples collected from the fortnightly collection. Clinical and demographical data were collected at routine study visits. Swabs were inoculated onto Mannitol Salt Agar (MSA) (National Health Laboratory Services, Green Point Media Laboratory Cape Town, South Africa) and incubated at 35°C for 18–24 h in room air. Mannitol-positive and DNase-positive isolates were identified as *S. aureus* (Kateete et al., 2010). *S. aureus* isolates were characterized by *spa*-typing and clustered based on their genetic relatedness into *spa*-clonal complexes (CCs) using the Based Upon Repeat Pattern (BURP) clustering algorithm of the Ridom Staph Type software (Ridom GmbH, Münster, Germany).

This study was approved by the Human Research Ethics Committee of the Faculty of Health Sciences, University of Cape Town (HREC ref: 401/2009 and 740/2013) and the Western Cape Provincial Child Health Research Committee. Written informed consent was obtained from parents and renewed annually.

### Statistical Analysis and Graphical Representation

An acquisition event was defined according to Miller et al. (2014) as a *S. aureus*-positive culture subsequent to two consecutive negative swab cultures, or the detection of a new *spa*-CC in a child with previously positive cultures of a different *spa*-CCs. Loss of *S. aureus* colonization was defined as two consecutive negative swabs for *S. aureus*, or two consecutive swabs without the previous *spa*-CC (Miller et al., 2014). The acquisition and loss events were estimated to occur at the midpoint between the last negative and the first positive swab, and between the last positive and first negative swab, respectively. The duration of colonization by a genotype (*spa*-CC) was measured as the time between acquisition and loss. The median permutation test was used to assess the significance in the difference between median carriage duration of individual genotypes. Recurrent acquisition episodes both at the species and the genotype levels were determined using the conditional gap-time model. The Cox proportional hazards model was used to investigate the determinants of time to first *S. aureus* acquisition; variables with *p*-values of <0.2 in the univariable models were included in a multivariable model.

The carrier index (the proportion of samples collected from each infant during the first year of life which were positive for *S. aureus*) was calculated in order to define *S. aureus* carriage patterns: persistent (carrier index ≥0.8), prolonged (carrier index ≥0.5), intermittent (0 < carrier index ≤0.5), and no carriage (carrier index = 0). Two genotype diversity measures were used: the absolute number of genotypes (*spa*-CCs) carried by each infant, and the Shannon-Weiner Diversity Index, a measure of alpha diversity (the richness and evenness of taxa within a single sample) (Shannon, 1948; Simpson, 1949). In this study, the alpha diversity was applied to measure the variation in carriage of different genotypes of each infant over the first year of life. Pearson’s correlation coefficient for the carrier index and both of the genotype diversity measures was estimated. Analysis was collectively performed in R version 3.1.2 (R Foundation for Statistical Computing, Vienna, Austria) (R Core Team, 2014).

## Results

The clinical and demographic characteristics of 137 mother-infant pairs are summarized in Table 1. The median gestational age was 39 weeks (Inter-quartile range (IQR) 38–39 weeks); 56% (77/137) of the infants were female. A total of 3417 infant NP swabs were collected. Swabs were collected 2-weekly (median 14 days; IQR 13–15) from each infant for the first year of life. The median number of NP swabs collected per infant was 24 (IQR; 23–26) with a minimum of 18 swabs. There were 125 maternal NP swabs obtained at the time of birth, of which 21/125 (17%) yielded *S. aureus* on culture. *S. aureus* was isolated from 21% (725/3417) of infant NP swabs. Overall, 88% (121/137) of infants acquired *S. aureus* at least once during their first year of life. Of 578 isolates BURP analysis clustered 71 different *spa*-types into 11 *spa*-CCs. Eleven of the 71 *spa*-types represented singletons (116 isolates) and three *spa*-types (27 isolates) were excluded from the cluster analysis due to the small number of repeat regions.

**Table 1:** Participant characteristics.

Characteristics (*n* = 137)	No. (%)
**Gender**	
Female	77 (56.2)
Gestational age: median (range; IQR)	39 (27-42; 38-39)
Premature birth (<37 weeks)	21 (15)
**Mode of delivery**	
Vaginal	109 (80)
Cesarean	28 (20)
**Baby feeding method during first 6 months**	
Exclusive breast feeding	38 (28)
Exclusive formula feeding	60 (44)
Mixed feeding	39 (28)
Day care attendance	81 (59)
HIV exposure	33 (24)
**Smoke exposure**	
Smoker father	84 (61)
Smoker mother	45 (34)
Both parents	25 (34)
Either parent smoker	95 (69)
Any smoker exposure	114 (83)
**Socioeconomic status**	
Low	74 (54)
Moderate-high	63 (45)
Animals in the house	62 (45)
**Maternal education**	
Primary	13 (10)
Secondary	115 (86)
Tertiary	6 (4)
Infants treated for TB	18 (13)
Household size >5	50 (36)
Number of people in household	5 (1-20; 3-6)
**Median (range; IQR)**	
Having a sibling younger than 5 yrs	47 (34)

*HIV, Human Immunodeficiency Virus; IQR, interquartile range; TB, Tuberculosis; yrs., years.*

### Incidence of *S. aureus* Acquisition at the Species and Genotype Levels

**FIGURE 1:**
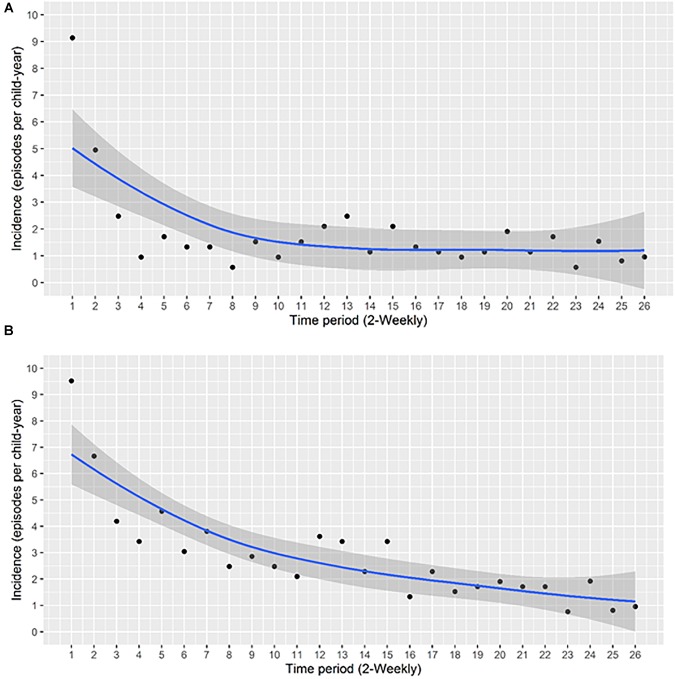
The incidence of acquisition of *S. aureus* NP carriage during the first year of life at the **(A)** species level and **(B)** at the genotype level. The incidence of acquisition per child-year at 2-weekly intervals was calculated. Each data point represents the incidence of acquisition over the period between the previous and the indicated time point. A smoothing function shown by the blue line, with a 95% confidence interval for the smoothing function (the area shaded in gray), was fitted.

A total of 248 acquisition events occurred during the study period, yielding an overall acquisition incidence of 1.83 episodes per child-year (95% CI 1.61–2.07). The highest incidence was observed during the first 2 weeks of age, with 9.2 episodes per child-year (95% CI 6.91–12.08) (Figure 1A).

At the genotype level, the total number of acquisition events was 390 with an overall acquisition incidence of 2.8 events per child-year (95% CI 2.6–3.1). The highest incidence occurred during the first 2 weeks of life, with 9.5 episodes per child-year (95% CI 7.2–12.5), thereafter, a gradual decrease was observed (Figure 1B). Overall, we noted higher acquisition incidence rates at the genotype level compared to the species level (Figure 1A,B).

**FIGURE 2:**
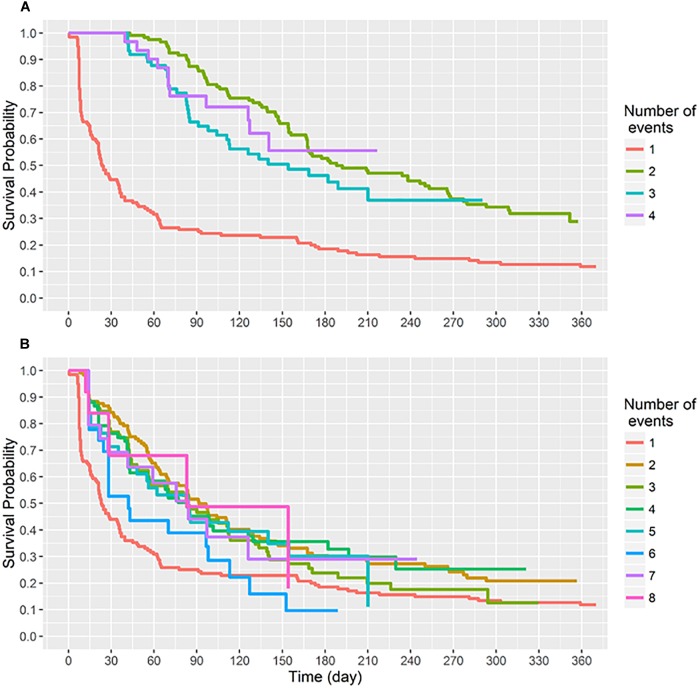
Survival time (days) to sequential *S. aureus* acquisition (1st, 2nd, 3rd, etc.) events. Recurring acquisition events both at **(A)** the species and **(B)** the genotype levels were assessed using the conditional gap-time model. **(A)** Acquisition events analyzed at the species level. Two infants had five acquisition events, these have been omitted. **(B)** Acquisition events analyzed at the genotype level. Two infants had nine acquisition events, these have been omitted.

**FIGURE 3:**
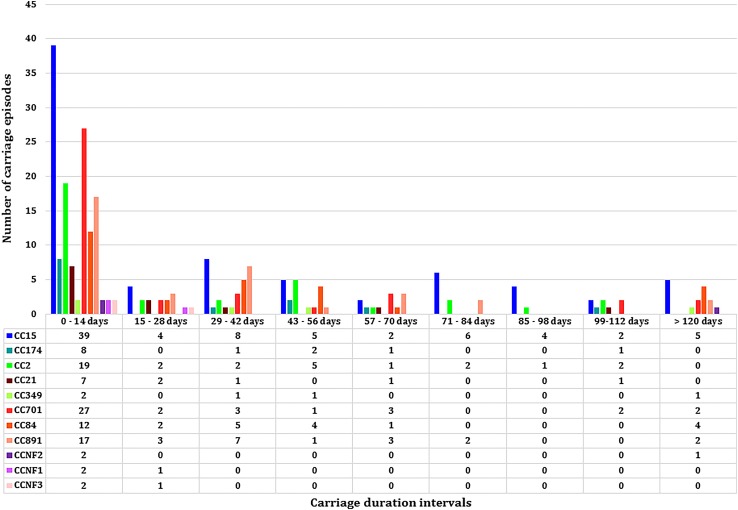
The median carriage duration of different genotypes (*spa*-clonal complexes, CC) stratified by different intervals of carriage.

By 91 days of age 75% of infants had acquired *S. aureus* for the first time (Figure 2A). First acquisition occurred at a median age of 23 days (95% CI 21–36 days). Of the 121 carrier infants, 64% (*n* = 77) acquired *S. aureus* for a second time at a median age of 188 days (95% CI, 168–266 days); 31% (*n* = 37) acquired *S. aureus* a third time, and 9% (*n* = 11) of carrier infants had four acquisition events during the first year of life (Figure 3). At the genotype level, 75% (91/121) of the infants who had a first acquisition, had a second acquisition event (either identical, related or a different *spa*-type) at a median age of 91.5 days (95% CI, 70.5–126.5 days) (Figure 2B). A total of nine new acquisition events at the genotype level occurred in two infants.

### Determinants of First Acquisition of *Staphylococcus aureus*

The Cox proportional hazards model was used to determine covariates associated with time to *S. aureus* first acquisition (Table 2). A greater number of individuals (≥5 individuals) living in the home [Hazard Ratio (HR) 1.06, 95% CI 1.07–1.43, *p* = 0.01], having a *S. aureus* carrier mother (HR; 1.5, 95% CI 1.2–2.47, *p* = 0.03), and infants having a positive TST at any time during the first year of life (HR; 1.81, 95% CI 0.97–3.3, *p* = 0.06) were all associated with earlier first *S. aureus* acquisition (Table 2). TST reactivity was significant only in the multivariable analysis, with no interaction observed with the number of individuals in the household.

**Table 2:** Univariable and multivariable analysis of factors associated with time to *S. aureus* first acquisition.

Factor	Hazard of time to first acquisition of *S. aureus*		
	HR	(95% confidence interval)	*p*-Value
**Univariable**			
HIV-exposure	0.92	0.60–1.39	0.70
Day care attendance	1.29	0.75–2.23	0.84
Preterm	0.94	0.62–1.42	0.78
Having an animal in the household	0.97	0.66–1.39	0.88
Smoking mother	0.84	0.57–1.23	0.38
Smoking father	0.82	0.56–1.18	0.29
More than 5 people in household	1.06	1.01–1.12	0.02
Delivery mode (Vaginal vs. cesarean)	0.80	0.44–1.47	0.48
Maternal *S. aureus* carriage	1.57	0.96–2.57	0.06
Infant hospital admission	0.74	0.34–1.63	0.46
Formula feeding at 6 months (vs. breast-feeding)	1.02	0.51–2.07	0.95
Mixed feeding at 6 months (vs. breast-feeding)	1.19	0.50–2.82	0.69
TB infection during the first year of life^∗^	1.29	0.75–2.2	0.24
Having younger sibling	0.75	0.49–1.14	0.18
Low-moderate SES	1.04	0.52–2.07	0.92
Moderate-high SES	2.20	0.85–5.67	0.10
**Multivariable**			
More than five people in household	1.06	1.07–1.43	0.01
TB infection during the first year of life^∗^	1.81	0.97–3.3	0.06
Maternal *S. aureus* carriage	1.5	1.2–2.47	0.03

*HR, hazard ratio; TB, tuberculosis; SES, socioeconomic status. ^∗^As defined by the tuberculin Skin Test reactivity results.*

### Defining Carriage Patterns Based on Carriage Duration, Carrier Index, and Genotypic Diversity

Eleven *spa*-CCs were identified, which were carried by infants for different lengths of time (Figure 3). A median carriage duration of 14 days (the minimum possible estimated duration, given the definitions used) was observed for eight out of the 11 *spa*-CCs: CC2 (IQR; 14–55 days), CC15 (IQR; 14–70 days), CC21 (IQR; 14–34.5 days), CC174 (IQR; 14–45 days), CC701 (IQR; 14–41 days), clonal complex with no-founder (CCNF) 1 (IQR; 14–28 days), CCNF2 (IQR; 14–126 days), and CCNF3 (IQR; 14–25 days). For the remaining four *spa*-CCs, longer median carriage duration was observed; 20.5 days for CC891 (IQR; 14–42 days), 29.5 days for CC84 (IQR; 14–56 days), and 42 days for CC349 (IQR; 14–52). Duration of carriage of *spa*-CC15, the most frequently detected *spa*-type, varied between 0 and 215 days. Using the median permutation test, the only significant difference in the carriage duration was observed between CC84 and CC701 (Table 3).

**Table 3:** Significance level for pairwise comparison of differences in median carriage duration for the 11 *spa*-CCs.

	CC2	CC15	CC21	CC84	CC174	CC346	CC701	CC891	CCNF1	CCNF2	CCNF3
CC2	NA	0.56	0.54	0.29	0.35	0.20	0.06	0.60	0.40	0.38	0.40
CC15		NA	0.63	0.19	0.46	0.18	0.18	0.70	0.46	0.47	0.46
CC21			NA	0.24	0.42	0.05	0.18	0.62	0.53	0.53	0.52
CC84				NA	0.47	0.65	**0.03**	0.54	0.25	0.43	0.30
CC174					NA	0.18	0.08	0.58	0.29	0.30	0.31
CC346						NA	0.07	0.17	0.11	0.48	0.12
CC701							NA	0.10	0.24	0.25	0.24
CC891								NA	0.64	0.43	0.60
CCNF1									NA	0.39	0.40
CCNF2										NA	0.40
CCNF3											NA

*CC; clonal complex, NF; no founder, NA; not applicable. Significant difference would be less than 0.05. The table shows the raw permutation values. No significant differences were observed between the median carriage durations for each of the *spa*-CCs except for CC701 and CC84 (in bold).*

**FIGURE 4:**
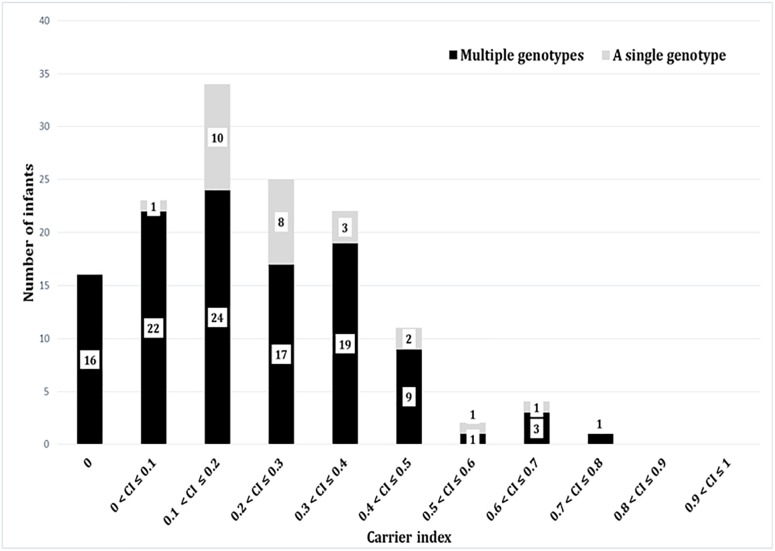
The carrier indices for 137 infants followed up longitudinally every 2 weeks for the first year of life. The carrier index is defined as the number of *S. aureus* positive swabs divided by the total number of NP swabs collected per infant during the study period. The gray bars represent number of infants with specified carrier index who carried a single genotype in all positive swabs, whilst dark bars represent number of infants who carried multiple different genotypes at different time points. For example, 34 infants had a carrier index of greater than 0.1 and less than or equal to 0.2. Of these 34 infants, 10 infants carried a single genotype over the period of study, whilst 24 carried multiple genotypes.

**FIGURE 5:**
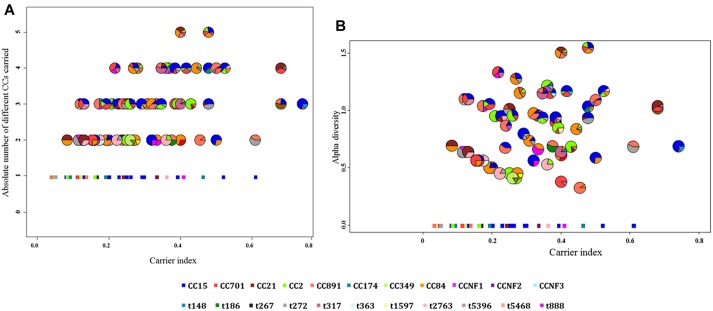
The correlation between the carrier index and **(A)** absolute number of *spa*-CC or **(B)** alpha diversity. **(A)** The carrier index vs. absolute number of *spa*-CC carried by each infant showing a positive correlation (*r* = 0.5, *p* < 0.0001). **(B)** The carrier index vs. alpha diversity showing a stronger positive correlation (*r* = 0.6, *p* < 0.0001). The carrier index was calculated as the proportion of *S. aureus* positive samples divided by the total number of samples collected for each infant. The alpha diversity as measured by the H-index reflects the number and distribution of genotypes carried by each infant. The minimum value for the absolute number of *spa*-CC is 1, while the minimum alpha diversity is zero. Each pie chart represents the proportions of different genotypes carried by a single infant throughout the first year. The square dots in both figures indicate infants who carried only a single genotype in all positive samples.

Based on the carrier index, 12% (16/137) of infants were non-carriers, while the rest of infants (121/137) had indices between 0.03 and 0.74 (Figure 4). Using a carrier index of 0.8 as a cut-off to distinguish between persistent and intermittent carriage, we observed no persistent carriers. Therefore, we elected to refer to infants with a carrier index of more than 0.5 as having “prolonged carriage.” Accordingly, 6% (7/121) of carrier infants had prolonged carriage of *S. aureus* at the species level. However, at the genotype level, only 2% (2/121) of infants had prolonged carriage with a single genotype (Figure 4). Overall, 39% (47/121) of infants carried only a single genotype during the first year, with a carrier index ranging from 0.03 to 0.61. A positive correlation (*r* = 0.5; 95% CI 0.35–0.62, *p* < 0.0001) was observed between the absolute number of different *spa*-CC carried by an infant and the carrier index (Figure 5A). The median alpha diversity observed was 1.3 (IQR; 1.03–1.52). A positive correlation was also observed between alpha diversity and carrier index (*r* = 0.6; 95% CI 0.46–0.70, *p* < 0.0001) (Figure 5B).

## Discussion

This study is unique in applying genotypic measures of strain diversity to describe the dynamics of *S. aureus* NP colonization in a frequently sampled cohort of African infants during the first year of life. Key findings include a high incidence of *S. aureus* acquisition at the genotype level, the absence of persistent carriage and a correlation between increased carriage frequency and measures of strain diversity. We also noted a high incidence of acquisition at the genotype level within the first 4 weeks of life. There was no clear association between strain genotype and carriage duration. TST reactivity was associated with reduced time to first acquisition of *S. aureus.*

*Staphylococcus aureus* carriage in our cohort was less common than that previously described in another African cohort study. By 30 days of age 45% of infants had acquired *S. aureus* for the first time, compared with 75% at the same age in The Gambia (Kwambana et al., 2011). A possible reason for the higher carriage rates among infants from The Gambia may be climate, as The Gambia has considerably higher annual temperatures. In a review, Leekha et al. (2012) report a trend toward increased *S. aureus* carriage and infections during the hot season.

Large family size (>5 individuals), and *S. aureus* maternal carriage were associated with reduced time to *S. aureus* first acquisition; these findings are in line with previous studies (Bourgeois-Nicolaos et al., 2010; Lebon et al., 2010; Soltani et al., 2014). Previous studies have used TST (induration reaction of >10 mm) as a measure of TB infection in settings where the Bacillus Calmette-Guerin (BCG) vaccine is administered (Martinez et al., 2018; Pelzer et al., 2018). We report an association between pediatric TB infection (as defined by TST) and a shorter time to first acquisition of *S. aureus*, possibly reflecting unmeasured exposures to both organisms, for example, exposure to health care facilities or antimicrobial therapy. This observation requires further investigation. Previous studies have shown that cigarette smoke exposure was negatively associated with *S. aureus* NP carriage, however, we did not observe any association between either maternal of paternal smoking and *S. aureus* first acquisition in infants (Chatzakis et al., 2011).

The observed high incidence rate early in life could not be explained by the large family size, maternal *S. aureus* carriage or TB infection. However, in previous studies, *S. aureus* carriage among healthy infants was shown to have a negative association with age (i.e., carriage decrease with increasing age). This could be explained by the microbial interference within the nasopharynx, where *S. aureus* is the predominant colonizer for the first 6 weeks namely and it gets replaced mainly by the *Streptococcus pneumonia*, *Haemophilus influenzae*, and *Moraxella catarrhalis* with increasing age (Biesbroek et al., 2014).

Twelve percent of infants in our study were non-carriers. None of the infants had a carrier index of 0.8 or more (previously used to define persistent carriage), at either the species or genotype level. This is in agreement with longitudinal studies from the United Kingdom and the Netherlands where persistent carriage was rarely found during infancy (Peacock et al., 2003; Lebon et al., 2008). However, in a study from Belgium which included 333 preschool children aged 3–6 years, 15% of the children were classified as persistent carriers based on consecutive carriage of the same genotype (Blumental et al., 2013).

A novel finding was that when combining the carrier index with measures of genotypic diversity, a higher carrier index was associated with increased genotypic diversity, i.e., infants with more frequent or prolonged carriage also harbored more genotype diversity. This observation is in contrast with adult studies where persistent carriers were observed to often carry the same genotype for prolonged periods (Hu et al., 1995; Van Belkum et al., 1997). For example, one study of 104 healthy adults, in which a median number of 14 swabs was collected over 19 months, showed that 14% of participants carried a single genotype with a carrier index of 0.9 or higher (Eriksen et al., 1995). In another study which involved 91 university staff and was conducted over 12 weeks, 36% had carrier indices of 0.8 or higher. Participants with carrier index of 1.0 were found to carry the same strain 8 years later (Vandenbergh et al., 1999). The NP microbiota in early life is highly unstable, and is repeatedly disturbed by intercurrent viral infections (van den Bergh et al., 2012; Biesbroek et al., 2014). This changing ecological environment may influence the ability of *S. aureus* strains to colonize the NP persistently, which might explain the high genotype diversity amongst infants, in contrast to adult studies.

The importance of including genotypic measures of strain diversity is demonstrated by our study. For example, seven infants carried *S. aureus* with a carrier index of greater than 0.5, but only two of them carried a single strain throughout the first year. There are few studies which have longitudinally investigated NP carriage of *S. aureus* at the genotype level among healthy infants (Peacock et al., 2003; Lebon et al., 2008). In a study of 100 healthy infants from the United Kingdom with nine NP swabs per infant over the first 6 months of life, 25% of infants had no detectable NP carriage, and most infants carried a single genotype regardless of whether the carriage pattern was persistent or intermittent (Peacock et al., 2003). In a birth cohort study in the Netherlands, three NP swabs were collected from 443 infants over 14 months, with only two swabs collected during the first year of life (at 1.5 and 6 months). All three swabs were negative for *S. aureus* in 36% of infants, while, only two infants carried a single strain in all three swabs (Lebon et al., 2008). Verhoeven et al. (2012) suggested that a total of seven swabs collected over 5 weeks are sufficient to distinguish persistent carriage from the other patterns. However, non-carriage and persistent carriage rates tend to decrease with increasing follow-up periods and decreasing culture intervals (Vandenbergh et al., 1999). In our study, a median of 24 NP swabs were collected per infant over 1 year, with a minimum of 18 swabs, compared to three and nine in the studies from the Netherlands and the United Kingdom, respectively. The intense 2-weekly sampling is a key and unique feature of this study; our data may provide a more accurate estimate of “true” non-carriage (in our study estimated at 12%) and persistent/prolonged carriage.

The carriage duration as well as the diversity of the genotypes carried by an individual are among the criteria used in previous studies to distinguish intermittent from persistent carriers; persistent *S. aureus* carriers tend to carry fewer strains for longer periods of time (Van Belkum et al., 2009; Blumental et al., 2013; Muthukrishnan et al., 2013). In this study, neither the carriage duration nor genotype diversity was able to segregate the infants into distinct groups. In fact, infants with prolonged carriage harbored more diverse genotypes. Although *spa*-CC15 was previously found to be less likely associated with long term compared to intermittent carriage (Miller et al., 2014), in this study no association between carriage duration and genotype was observed.

Limitations of our study include that we did not specifically look for co-carriage with multiple strains in the same individual, and infants were studied for a only 1 year. However, this study represents one of the most intensely sampled cohorts reported. Extended follow up is currently underway to identify when children first develop true prolonged carriage with the same strain, as has been observed in adults. Strengths of our study include the longitudinal nature of the study, allied to the comprehensive epidemiological data collection and strain typing, which allowed for detailed analysis of colonization dynamics at the strain level. The approach of combining genotype diversity and carrier index may be useful for future studies describing the epidemiology of colonization with *S. aureus*.

## Conclusion

In conclusion, we genotyped *S. aureus* isolates from the NP of intensively sampled infants from South Africa to extend our understanding of carriage dynamics in this age group. Incorporating genotypic measures allowed us to obtain a more accurate assessment of persistent or prolonged carriage. Persistent carriage was not identified, while prolonged carriage, defined by a carrier index of >0.5, was uncommon. *S. aureus* NP carriage is highly dynamic in infants. Increased carrier index was associated with genotype diversity, and there was no clear association between particular genotypes and duration of carriage.

## Author Contributions

HZ was the principal investigator of the Drakenstein Child Health Study and conceived and designed the birth cohort study together with MN. SA and MN conceptualized the *S. aureus* study. LR contributed to the analysis and interpretation of results and development of the manuscript. JR, PN, SG-L, and FD assisted with the statistical analysis and graphical representation. SA drafted this manuscript. All authors reviewed and improved the final version of the manuscript.

## Conflict of Interest Statement

The authors declare that the research was conducted in the absence of any commercial or financial relationships that could be construed as a potential conflict of interest.
